# An integrated nutrition and health program package on IYCN improves breastfeeding but not complementary feeding and nutritional status in rural northern India: A quasi-experimental randomized longitudinal study

**DOI:** 10.1371/journal.pone.0185030

**Published:** 2017-09-20

**Authors:** Veena Singh, Saifuddin Ahmed, Michele L. Dreyfuss, Usha Kiran, Deepika N. Chaudhery, Vinod K. Srivastava, Ramesh C. Ahuja, Abdullah H. Baqui, Gary L. Darmstadt, Mathuram Santosham, Keith P. West

**Affiliations:** 1 Center for Human Nutrition, Department of International Health, Johns Hopkins Bloomberg School of Public Health, Baltimore, Maryland, United States of America; 2 Department of Population and Family Health, Johns Hopkins Bloomberg School of Public Health, Baltimore, Maryland, United States of America; 3 CARE-India, New Delhi, India; 4 King George Medical University, Lucknow, India; 5 Health Systems Division, Department of International Health, Johns Hopkins Bloomberg School of Public Health, Baltimore, Maryland, United States of America; TNO, NETHERLANDS

## Abstract

**Background:**

Undernutrition below two years of age remains a major public health problem in India. We conducted an evaluation of an integrated nutrition and health program that aimed to improve nutritional status of young children by improving breast and complementary feeding practices over that offered by the Government of India’s standard nutrition and health care program.

**Methods:**

In Uttar Pradesh state, through multi-stage cluster random sampling, 81 villages in an intervention district and 84 villages in a comparison district were selected. A cohort of 957 third trimester pregnant women identified during house-to-house surveys was enrolled and, following childbirth, mother-child dyads were followed every three months from birth to 18 months of age. The primary outcomes were improvements in weight-for-age and length-for-age z scores, with improved breastfeeding and complementary feeding practices as intermediate outcomes.

**Findings:**

Optimal breastfeeding practices were higher among women in intervention than comparison areas, including initiating breastfeeding within one hour of delivery (17.4% vs. 2.7%, p<0.001), feeding colostrum (34.7% vs. 8.4%, p<0.001), avoiding pre-lacteals (19.6% vs. 2.1%, p<0.001) and exclusively breastfeeding up to 6 months (24.1% vs. 15.3%, p = 0.001). However, differences were few and mixed between study arms with respect to complementary feeding practices. The mean weight-for-age z-score was higher at 9 months (-2.1 vs. -2.4, p = 0.0026) and the prevalence of underweight status was lower at 12 months (58.5% vs. 69.3%, p = 0.047) among intervention children. The prevalence of stunting was similar between study arms at all ages. Coefficients to show the differences between the intervention and comparison districts (0.13 cm/mo) suggested significant faster linear growth among intervention district infants at earlier ages (0–5 months).

**Interpretation:**

Mothers participating in the intervention district were more likely to follow optimal breast, although not complementary feeding practices. The program modestly improved linear growth in earlier age and weight gain in late infancy. Comprehensive nutrition and health interventions are complex; the implementation strategies need careful examination to improve feeding practices and thus impact growth.

**Trial registration:**

The trial was registered with ClinicalTrials.gov, NCT00198835.

## Introduction

Undernutrition among children in developing countries remains a significant public health problem. Most recent estimates based on WHO Child Growth Standards suggest that 41% of the under-five children in the United Nations’ South-Central Asian Region are stunted and 33% are underweight [1]. India alone, with prevalence rates of 48% and 43%, has an estimated 54.3 million stunted and 48.2 million underweight preschool aged children [2, 3], representing 31% and 43% of all the developing world's burden, respectively. Undernutrition has also been associated with increased risk of all-cause mortality, as well as fatality due to measles, malaria, pneumonia and diarrhea [4].

Small-scale, nutrition education interventions have registered successes in increasing total caloric intake, energy density and intake from complementary foods [5–9], yet effects on growth during the first two years of life have been mixed [5–8, 10–12]. A recent efficacy trial, after twelve-month intervention significantly increased complementary food intake and reduced stunting in complementary feeding group but not in the complementary feeding + play group [12]. Given a need to move beyond small scale or efficacy trials have been realized, in India, there have been only a few evaluations of large-scale community-based nutrition interventions in the last decade, which have also been limited in scope. Few evaluations have employed prospective, comparison group designs, leaving this area of implementation science lacking in high quality impact data [13–15].

In India, nutrition and health services are delivered by the Government of India’s (GOI) Integrated Child Development Services (ICDS) program under the Department of Child Development, Ministry of Women and Child Development and the Reproductive and Child Health (RCH) Department of the Ministry of Health & Family Welfare (MOHFW). Anganwadi workers (AWW), community-based health workers appointed under the ICDS program, are responsible for providing nutrition education, supplementary foods and micronutrient supplements in coordination with more highly trained, but fewer-in-number auxiliary nurse midwives (ANM). AWWs also assist the ANMs in identifying children and families in need of program participation. The ANM is responsible for delivering ante- and post-natal care that includes iron and folic acid supplementation, attending child birth, providing immunizations and periodically distributing vitamin A to pre-school children.

We prospectively evaluated an enhanced version of the ICDS and RCH service package delivered by the GOI in the State of Uttar Pradesh, called the Integrated Nutrition and Health Program (INHP II). The INHP II aims to improve growth under two years of age by targeting mothers during pregnancy through post-natal lactation periods with nutrition services facilitated by an international NGO, CARE-India. Changes in feeding practices, nutritional status and growth were compared to outcomes achieved within an ICDS “basic” services area, which received usual governmental services, within the same state.

## Subjects and methods

### Evaluation design

**Fig 1:**
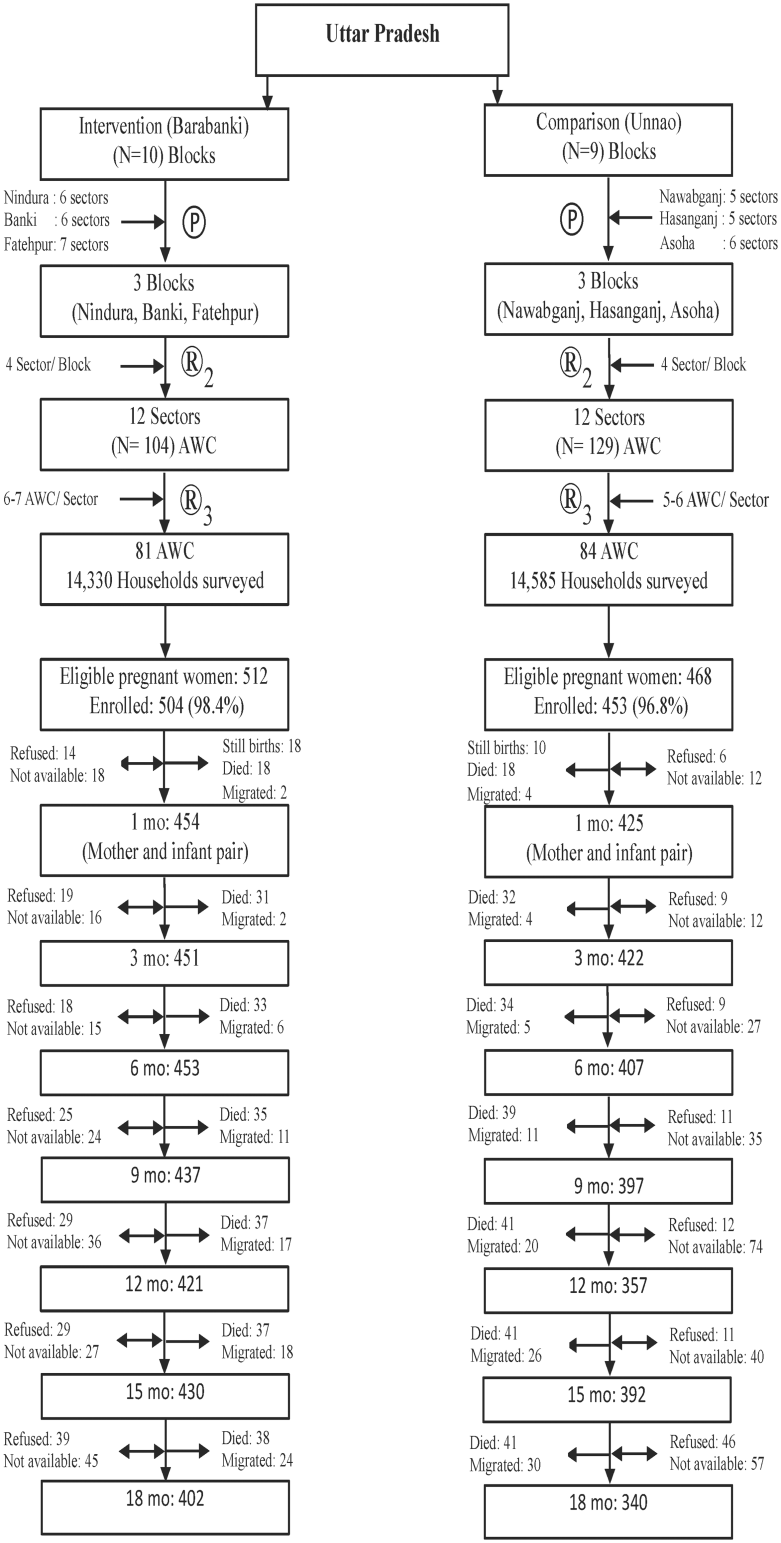
Flow chart of subject selection and follow-up*. * Still births, died, migrated are cumulative numbers ℗ Purposively selected blocks based on geographical location due to cohort nature of the study ^®^ Random selection of sectors and AWCs → Single arrow demonstrates permanent removal from the subsequent visits due to death or migration ↔ Double arrow shows refusal or ‘not available’ and may or may not be a part of subsequent visits ‘Not available’ were not at home at the time of scheduled visits. They were visited twice for two subsequent weeks after first scheduled visit before assigning this status for the visit.

A prospective, cohort assessment was designed and implemented, whereby in each of the intervention and comparison district sites pregnant women were identified, enrolled, provided program inputs and followed with their infants through 18 months after child birth. Within each district, a multi-stage, pseudo-random sampling design was employed to identify AWCs, by first purposively selecting three rural blocks based on physical proximity to other selected blocks and the state capital (project office). Additional criteria were the absence of other major maternal and child health projects and comparability with respect to population size and number of AWCs. Further, in the intervention district, only blocks where CARE-India's INHP-II program was active (10 of 15 blocks) were eligible. By these joint criteria, Blocks Nindura, Banki and Fatehpur in the intervention and Nawabganj, Hasanganj and Asoha in the comparison district were selected. Each block in both districts consisted of 5–7 sectors. From each of the three selected blocks in each district, 4 sectors were randomly selected (12 per district, 24 in all). AWCs in these sectors were enumerated, leading to 135 and 192 AWCs in the intervention and comparison districts, respectively, being enlisted. In the final sampling stage, 5–7 AWCs were randomly sampled per sector from both the districts to achieve a final sample of 81 and 84 AWCs in Barabanki and Unnao Districts, respectively, expected to yield 400 or more third trimester pregnant women in each district who would be eligible for enrollment into the evaluation (Fig 1).

District program officers and chief medical officers were contacted before starting the study to fully brief them about the study and obtain approvals. Village heads were contacted for oral permission and written informed consent was obtained from each study participant.

Randomization was done using computer generated random numbers by a co-investigators, who was not engaged with the data collection activity on ground, at the Johns Hopkins University, Bloomberg School of Public Health (JHBSPH), USA. Chief Medical officers, District program officers, primary service providers (ANMs & AWW), and study participants were not aware of the ‘basic’ and ‘CARE-enhanced’ nature of the intervention. The teams of two staff members, three teams per districts were swapped every two months to avoid any data collection bias.

The institutional review board at the Johns Hopkins Bloomberg School of Public Health (JHBSPH), Baltimore, MD and the ethical committee at King Georges Medical University (KGMU), Lucknow, UP approved the study. This was an ‘exempt’ trial that was not a "clinical trials" that require clinicaltrials.gov registration. The initial IRB approval from the JHBSPH was obtained on August 1, 2003. In 2005, the International Committee of Medical Journal Editors (ICMJE) began to require trial registration as a condition of publication. Therefore, to get this registered, IRB approval from the KGMU was obtained on December 28, 2004 and the trial was registered in 2005. The authors confirm that all ongoing and related trials for this drug/intervention are registered.

Collaborative investigators at The Johns Hopkins Bloomberg School of Public Health (JHBSPH) and the King Georges Medical University (KGMU), India, designed the evaluation, developed research instruments, and trained staff who collected, entered and managed the data. The study is registered with ClinicalTrials.gov, NCT00198835.

## Study setting

**Fig 2:**
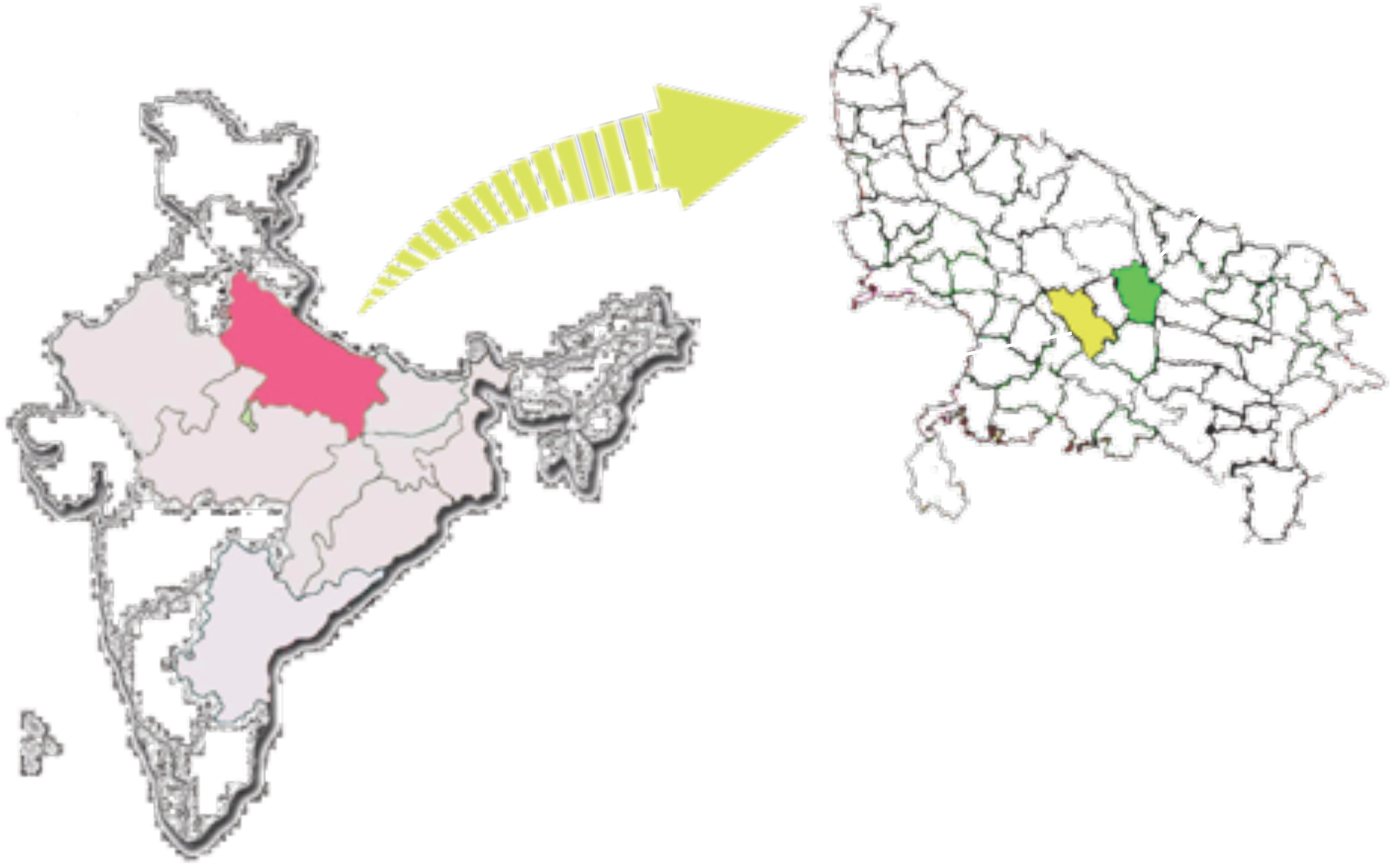
Locations of Barabanki (green) and Unnao (yellow) study districts in Uttar Pradesh (pink). Lightly shaded states show locations of CARE-India’s INHP II programs.

The study was carried out in the state of Uttar Pradesh, India’s most populous state. With 16.2% of the country’s population (166.2 million), a density of 689 persons (versus a national average of 312) per sq. km, and an underweight prevalence of 42.4%, the state has an estimated 9 million undernourished preschool children [2]. Approximately 44.2% of men and 65.9% of women are illiterate and 31.2% of the population subsists below the country's poverty line^3^. Within Uttar Pradesh, the site for the INHP-II intervention was Barabanki District and that for standard, basic ICDS services (comparison area), Unnao District (Fig 2).

### ICDS ‘basic’ program

Launched in 1975 by the GOI, ICDS is the world’s largest early childhood development program, seeking to reduce malnutrition, morbidity and mortality, and improve learning capacity, through improved village-based health and nutrition services delivered through Anganwadi centers (AWCs). Through 6,719 district projects, ICDS operates 1.24 million AWCs, serving the needs of approximately 92 million mothers and children each year [16].

#### CARE-India’s Integrated Nutrition and Health Program (INHP II)

The INHP II approach: The INHP II adopted a demonstration and replication approach for scaling-up select best practices through existing government system. Ten percent of the AWCs in the intervention districts were selected as demonstration sites. These sites were used to innovate and develop ‘best practices’ that could help solve operational problems of the ICDS and RCH programs. Here, INHP II worked with local NGOs and community-based organizations (CBOs), like Self-Help Groups, Panchayati Raj institutions (PRIs), academic and research institutions, private sector service providers, social marketing agencies, corporate social initiatives, professional bodies and motivated individuals and professionals to complement and support the efforts of the government systems. The practices deemed effective in solving oprational problems and generate evidence in convincing the decision maker were taken to scale, in remaining ninety percent of the replication sites.

In replication sites, the INHP II was mainly delivered using GOIs existing systems and ICDS and RCH functionaries. CARE placed 2–3 full-time professionals per district to support the ICDS and RCH programs at district and sub-district levels to improve implementation of the package of services delivered by the ANMs and AWWs. These CARE personnel acted as an external catalyst and facilitator for making these programs sharper, more focused and effective by providing the necessary technical, managerial and resource support at all critical levels of implementation. The evaluation was conducted at the replication sites.

The INHP II worked with the GOI's ICDS and RCH program to strengthen the quality and coverage of a package of health and nutrition services during pregnancy and the first two years of age. These include antenatal care, iron-folic acid (IFA) and vitamin A supplementation, immunization, community-based newborn care, and promotion of age-appropriate breast and complementary feeding practices (for children 6 to 24 months with regard to timing, quantity, frequency, quality; with an emphasis on responsive feeding and feeding during and after illness) [17] (Table 1).

**Table 1:** Age-specific breastfeeding and complementary feeding recommendations of CARE-India’s INHP II program^1^.

Age-ranges	Infant feeding recommendations
Breastfeeding	
At birth	Initiate breastfeed ≤1hr of birth Only breastfeed; do not give water, other fluids, honey water, *ghutti*, or food Only breastfeed up to 6 months of age
6–24 mo	Continue frequent, on-demand breastfeeding
Complementary feeding	
At 6 mo	Introduce home foods by feeding1-2 teaspoons at a time to gradually increasing the quantity and consistency
6–8 mo	Feed soft mashed home foods at least a *katori* (100 gm) 2 times per day Add 1/4 to 1/2 teaspoon of oil in each meal Feed variety of food and encourage the child to eat more
9–11 mo	Feed soft mashed home foods at least a *katori* (100 gm) 3 times per day Add 1/2 teaspoon of oil in each meal. Give curd, eggs, meat, fish, if available In addition give seasonal fruits such as papaya, mango, banana etc; encourage the child to eat more
12–24 mo	Feed soft mashed or in small quantities home foods at least a *katori* (100 gm) 4 times per day Add 1 to 2 teaspoon of oil in each meal. Give curd, eggs, meat, fish, if available Feed variety of food and encourage the child to eat more

^1^ Based on UNICEF/WHO breastfeeding and complementary feeding guidelines.

AWWs and ANMs were trained to impart age-specific advice about breastfeeding at birth, including the importance of initiating breastfeeding within 1 hour of childbirth, and withholding all other fluids or foods until 6 months of age. Workers were trained to encourage mothers to breastfeed frequently, on-demand, day and night, and increase the frequency of breastfeeding during and after illness. For children 6–24 months of age, workers advised mothers to continue frequent, on-demand breastfeeding, and to increase the frequency of breastfeeding during illness.

AWWs and ANMs were also trained to advise the child caregivers to introduce small amounts of home foods at age 6 months, gradually increasing the quantity and consistency after a few days such that, by 6–8 months of age, mothers were advised to give infants soft, mashed or thick, pasty home foods at least twice a day, increasing the frequency to ≥ 3 times a day by 9–11 months, and ≥ 4 times a day by 12–24 months of age; and to increase the total quantity of food consumed by their infants, from ≥ 200 gm by 6–8 months to ≥ 300 gm by 9–11 months of age and ≥ 400 gm by 12–24 months of age. Staff were trained to advise that younger infants (6–8 months) be fed rice mixed with *dal*, *khichr*i, *dalia*, rice *kheer* or *suji kheer* in addition to small amounts of cooked potato, carrot or egg/meat/fish, mashed banana or papaya. Mothers of children 9–24 months were advised to feed them rice, *dal*, mashed *chapati* in *dal* with vegetables (potatoes, carrots, pumpkin, etc.), green leafy vegetables, curd, eggs, meat and fish, when available. In addition, AWWs and ANMs were to advise that seasonal fruits such as papaya, mango and banana be given to children. To increase the caloric density of food, AWWs and ANMs were guided to advise caregivers to add initially 1/4 to 1/2 teaspoon of oil to food before serving, increasing the amount to ½ teaspoon by 9–11 months and 1–2 teaspoons by 12–24 months of age.

Staff members were trained to advise caregivers to interact with their children during feeding, provide meals in a separate bowl to monitor portions consumed, and use gentle persuasion, with stories, songs or toys, to keep the baby’s attention. After illness, AWWs and ANMs were trained to advise caregivers to increase the frequency by one additional time and quantity by 100 gm per feeding until weight gain resumes.

The presence of additional full-time professionals for supportive supervision with respect to visitng schedule, specifity of feeding advise with respect to content of food to be given at specific ages and emphasis on timing and frequency of visits were the distingiushing characteristics of the CARE ‘enhanced’ package.

## Sample size and data adequacy

Sample size was calculated to detect proportional differences in infant nutritional status at 6, 12 and 18 months of age between the intervention and comparison districts. Prevalence estimates from NFHS-3, Uttar Pradesh, India 2005–06 report The statistical power used for the calculations was 80% to detect a difference of 10–15% in stunting and underweight at a significance level of 95% (alpha = 0.05). To test the hypothesis of inequality of proportion of outcomes between exposure groups, the following formula was used for sample size calculation:

$$n=\frac{{\left(Z_{\frac{\alpha }{2}}\;\sqrt{2pq}+Z_{\beta }\sqrt{p1q1+p2q2}\right)}^{2}}{\Delta^{2}}$$

*where*,

*n* = *sample size in exposed and unexposed*

*Z_α/2_* = *1.96*

*Z_β_* = *0.84*

*p* = *Average proportion under the null*

*p_1_* = *proportion in unexposed group*

*p_2_* = *proportion in exposed group*

Δ = *minimum detectable difference (p_1_—p_2_)*

This sample size was also adequate for 50 kcal/day (SD: 200Kcal/day) in energy intakes from complementary foods at 7–10 months and 12–15 months of age at a significance level of 95% (alpha = 0.05). To enroll ~400 third trimester pregnant women in each district, 81–84 AWCs were targeted to identify ~1000 third trimester pregnant women with a 20% margin for refusals and losses to follow-up.

### Data collection

Field data collection was carried out by three teams per district, each comprised of two staff members. First, houses were listed in each of the 81 and 84 AWC catchment areas in the intervention and comparison districts, respectively. Between May 2004 and August 2004, resident women reporting to field investigators to be in their third trimester of pregnancy and intending to stay throughout the planned study time period, were identified, consented and enrolled into the study. These women were contacted 7 days after delivery and mother-child pairs were followed-up every three months from birth until the child reached 18 months of age or had died, or until the mother refused to further participate, the household permanently moved or the study ended on June 30, 2006.

Socio-demographic data were collected and a mother’s height and weight were measured at the time of third trimester enlistment. Data related to initiation of breast and colostrum feeding, avoidance of prelacteals and duration of breastfeeding were collected at the first postnatal home-visit (up to 1 month of age). Subsequently, breastfeeding and semi-quantitative, 24-hr recall data on variety, quantity and frequency of complementary feeding was assessed. At every postnatal visit child length and weight measurements were taken on a locally made, standardized infantometer and solar-powered UNICEF Seca floor scale (Model: 890, Scale mother/child electronic, Seca Ltd., Birmingham, UK), respectively, and converted into z-scores as per growth standards of the World Health Organization [18].

The primary impact outcome measure was improvement in the nutritional status and growth of children, reflected by length-for-age (LAZ) and weight-for-age (WAZ) z-scores. Improved adherence to advised breast and complementary feeding practices were intermediary, process outcomes that were measured as the percentage of children reported to be receiving optimal breastfeeding and complementary feeding for their age, based on a 24-hour dietary recall at each visit.

## Data quality control, review and analysis

Data quality control for fieldwork included unannounced visits by study field supervisors for 5% of all scheduled home visits in both areas. Completed data forms were edited in the field and transmitted to the project office in Lucknow for further editing and data entry. Data records were checked for completion, range, skip-pattern matching, evidence of heaping and consistency. Discrepancies were cross-checked with paper questionnaires for immediate resolution, when possible. Frequency distributions of the sample characteristics were examined for outliers, and derived variables evaluated (e.g., socioeconomic index quintile with principal component analysis, z-score distributions for reasonableness).

The economic status of the households were assessed with a wealth index, which was calculated using principal component analysis based on 16 household durable assets and housing characteristics including type of house, source of light, source of fuel, source of drinking water, type of toilet facility and number of rooms in the household. The wealth index was divided into five quintiles from level 1 (poorest) to level 5 (wealthiest). Cross-sectional differences between intervention and comparison districts were evaluated using Student’s t-test for continuous and Pearson’s chi-square for categorical variables. Unadjusted and adjusted odds ratios for dichotomized outcome variables were calculated to assess differences in risk.

The growth trajectory curves for length and weight by age were used to finalize cubic spline models with the knots at 6 and 18 months of age. To model growth, a random coefficient mixed model was used, allowing random intercept as well as slope for respective ages. The variables age and anganwadi centers were included as random effects to account for the clusterization of responses. Maximum likelihood estimates were used with unstructured option as random effects are correlated due to repeated measures to model variance that differs by age.

The analysis of the effects of intervention on physical growth was based on a latent growth model (Diggle et al., 1994) in which the outcome examined was the change in nutritional status (length and/or weight) over a one month period in interaction with the district of residence. Similar analyses were performed for the effects of gender, program and socio-demographic factors on physical growth. Mixed-effect multiple linear regression modeling allowed to adjust for the confounding factors among the socio-demographic characteristics of the sample. Age modeled with cubic spline terms allowed for three different coefficients for the ages 0–5 months, 6–17 months and > = 18 months of age. To obtain annual growth we summed the interaction terms after multiplying for respective number of months contributed. For example, to obtain an average one-year effect on length: [(age1 * district *6) + (age2 * district*6)]. All analyses were conducted with Stata/SE 11.1 (Stata Corp, TX).

## Results

A total of 28,915 households were surveyed, with 1,330 and 1,217 pregnant women identified in the intervention and the comparison districts, respectively (Fig 1). Of these, 512 in the intervention and 468 in the comparison districts were in their third trimester of pregnancy, of whom 504 (98.4%) and 453 (96.8%) agreed to participate in the evaluation, respectively. At the 18-month postnatal follow-up, data were collected on 420 (83.3%) and 352 (77.7%) mother-child pairs in the intervention and comparison districts.

The two study groups were similar in background characteristics, with some differences (Table 2). Literacy was low; two-thirds of women (67.6% vs. 69.1%) had never been to school and 7.8% and 6% had achieved a high school or higher education in the intervention and comparison groups, respectively. Fewer women in the intervention than comparison group worked outside the home (31.4% vs. 39.5%; p = 0.009), with most in both groups working in agricultural labor. Most of the deliveries took place at home (81.2% vs. 91%, respectively, p<0.001); more male children were born in both districts (53.4% and 56.4%; p = 0.361). There were differences in religion and in caste, and mothers in the intervention district were relatively older (<20 years: 16.1% vs. 20.5%; p = 0.016); however, there was no difference in the number of children women had (p = 0.938) or the birth interval between the last two live births (p = 0.650). There were also no differences in the mother’s mean gestational age at the time of enrollment or in anthropometric measures (Table 2).

**Table 2:** Background characteristics of study participants at the time of enrollment during the third trimester of pregnancy in Uttar Pradesh, India (2004).

Variable description	Project Districts		p-value^1^
	Intervention (Barabanki) (n = 504)	Comparison (Unnao) (n = 453)	
Socio-demographic characteristics			
Wealth index quintiles % ^2^			
1	19.2	21.0	0.344
2	18.2	22.2	
3	19.9	20.0	
4	20.7	19.3	
5	22.0	17.6	
Maternal education %			
Illiterate	67.6	69.1	0.427
1–7 yr	15.5	13.7	
8–9 yr	9.2	11.3	
10+ yr	7.8	6.0	
Paternal education %			
Illiterate	38.0	32.4	0.207
1–7 yr	16.7	20.9	
8–9 yr	24.1	25.6	
10+ yr	21.3	21.1	
Antenatal care check-up %	42.3	31.3	0.001
Place of delivery			
Home/other place	81.2	91.0	<0.0001
Government/private hospital	18.8	9.0	
Maternal Religion %			
Hindu	76.2	94.5	<0.0001
Muslim	23.8	5.5	
Caste %			
SC/ST	36.9	43.1	0.014
OBC	50.0	40.6	
General/others	13.1	16.3	
Physiological characteristics			
Maternal age %	(n = 500)	(n = 451)	
<20yr	16.1	20.5	0.016
20-34yr	75.9	75.3	
35-49yr	8.0	4.2	
Gravidity % ^3^			
1	19.0	18.1	0.938
2–3	15.6	16.8	
4–5	33.1	33.8	
6+	32.3	31.4	
Birth interval %^4^	(n = 315)	(n = 273)	
<24m	30.5	33.0	0.650
24-35m	36.5	33.0	
36+	33.0	34.1	
Infant gender %			
Male	53.4	56.4	0.361
Women’s gestational age (at the time of enrollment)			
In weeks (mean ± SD)	32.0 ± 3.9	31.5 ± 4.4	0.0907
Pre-term births % ^5^	16.8	12.9	0.091
Birth weight (Kg)	3.04 ± 0.31	3.13 ± 0.42	0.0708
Maternal weight (mean ± SD)	47.0 ±6.4	47.5 ±6.4	0.2508
Maternal height (mean ± SD)	149.1±5.7	149.3±5.4	0.7446

^1^ Statistical testing by chi square for contingency presentations, with r-1 x c-1 degrees of freedom where r = number of comparison groups (r = 2) and c = number of strata for each comparison (c = x) or by t-test for comparing continuous distributions, significant at:

*p<0.05.

**p<0.01 and

***p<0.001.

^2^ Wealth index was calculated using principal component analysis that include type of house, source of drinking water, toilet facility, room variables and ownership of 16 household items.

^3^ Applies to all pregnancy including current, pregnancy losses, still births and live births

^4^ Applies only to women with 2 or more previous live births whereby the ‘birth interval’ is the number of months between the last two live births, excluding the current pregnancy and any potential pregnancy loss between the 2 last live births

^5^ Calculated from last menstrual period (LMP) recall as reported by the mother at the time of enrollment and infant’s date of birth

### Impact on breastfeeding practices

Although nearly all the women in both districts reported breastfeeding their newborns (98–99%), initiation of breastfeeding within one hour of delivery was overall low (Table 3). However, early initiation was more frequently reported in the intervention arm (17.4% vs. 2.7%; p<0.001). In the intervention group, 34.7% of the women reported giving colostrum to their babies versus 8.4% in the comparison district (p<0.001). One-fifth (19.6%) of women reported avoiding giving prelacteals to their newborns in the intervention area, whereas only 2.1% did so in the comparison area (p<0.001). Exclusivity of breastfeeding was ascertained through 24-hour recalls at <1 month, 3 months and 6 months of age. Exclusive breastfeeding rates were significantly higher in the intervention than in the comparison area at each of these points in time.

**Table 3:** Breastfeeding practice outcomes upto 6 months of age based on 24 hour recalls collected at different ages.

Variable description	Project Districts		p-value^2^
	Intervention (Barabanki) (n = 492)^1^	Comparison (Unnao) (n = 450)^1^	
Breastfeeding initiation practices			
Initiation of breastfeeding<1hr %	17.4	2.7	<0.0001
Colostrum feeding %	34.7	8.4	<0.0001
Avoiding prelacteals %	19.6	2.1	<0.0001
Exclusive breastfeeding practices			
At 1 mo			
Exclusive % ^3^	74.1	66.4	<0.01
Pre-dominant % ^4^	12.4	11.9	
Partial % ^5^	13.5	21.7	
At 3 mo			
Exclusive %	52.6	42.5	0.01
Pre-dominant %	16.4	20.5	
Partial %	31.0	37.0	
At 6 mo			
Exclusive %	24.1	15.3	<0.01
Pre-dominant %	21.2	26.2	
Partial %	54.7	58.5	

^1^ 12 and 3 study participants from the intervention (492) and the comparison (450) districts were excluded due to refusal for all the successive visits

^2^ Statistical testing by chi square for contingency presentations, with r-1 x c-1 degrees of freedom where r = number of comparison groups (r = 2) and c = number of strata for each comparison (c = x), significant at:

*p<0.05.

**p<0.01 and

***p<0.001.

^3^ Exclusive breastfeeding include only feeding breastmilk

^4^ Predominant feeding include water, jeera, pudina, honey, sugar, jaggary, juice, tea, coffee or any other watery liquids

^5^ Partial feeding where mother reported giving any or all of the food items that include powdered milk, baby formula, buffalo, cow, goat milk, solid and/or semi-solid foods

### Impact on complementary feeding practices

**Fig 3:**
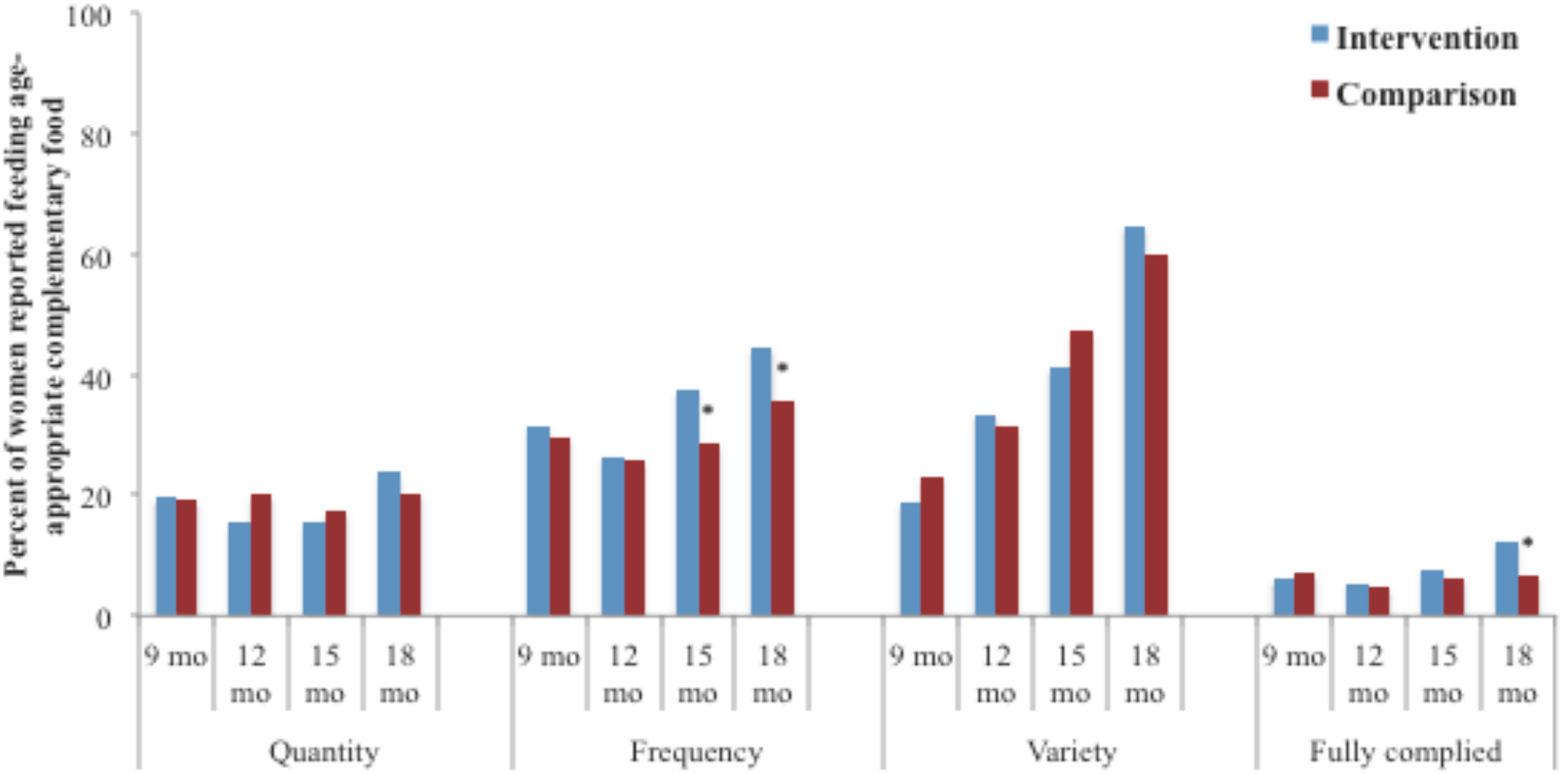
Complementary feeding practice outcomes from 9 to 18 months of age based on 24-hour recalls collected at different ages. * Statistical testing result comparing intervention and comparison districts by chi square for contingency presentations, with r-1 x c-1 degrees of freedom where r = number of comparison groups (r = 2) and c = number of strata for each comparison (c = x), significant at: *p<0.05. **p<0.01 and ***p<0.001 ^1^ Reported consumption of at least 300gm of solid or semi-solid food at 9 mo and at least 400gm of food at 12, 15 and 18 mo visits ^2^ Reported consumption of solid or semi-solid food at least 3 or more times at 9 mo and at least 4 or more times at 12, 15 and 18 mo visits ^3^ Estimated on the basis of reported consumption of 3 or more food groups ^4^ Estimated on the basis of reported consumption using all the three indicators of age-appropriate complementary feeding, i.e., frequency and variety

At 6 months of age, nearly half of the women in both districts reported giving solid and semi-solid foods to their infants (47.2% vs. 46.4%; p = 0.831). The 24-hour dietary recalls collected from 9–18 months demonstrated no significant difference between the intervention and the comparison districts in reported feeding of age-appropriate quantities of food to their children (Fig 3). The trend suggests improvement in total quantity of food given in the intervention area from 12–18 months, whereas, no such increase in the comparison district was observed. More women in the intervention district reported feeding their children at recommended frequencies at 15 months (37.4% vs. 28.7%; p = 0.038) and 18 months (44.4% vs. 35.8%; p = 0.027) of age, although there were no differences during the first year of life. Full compliance with recommended complementary feeding practices was low (<10%) in both districts, but was significantly higher in the intervention area (12.4% vs. 6.7%; p<0.01) at 18 months of age.

**Fig 4:**
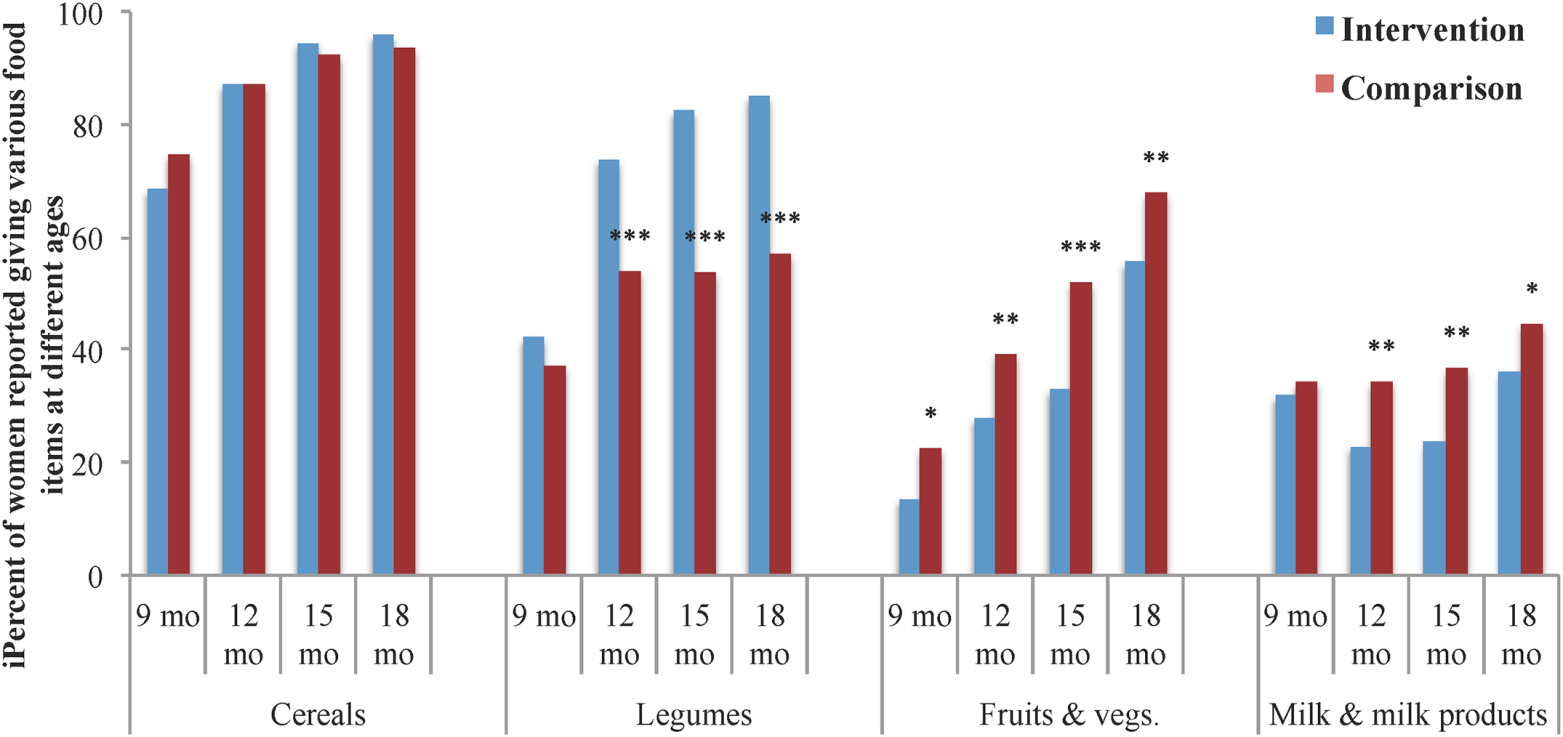
Variety of complementary food being consumed based on 24-hour recall at different ages. ^1^ Statistical testing result comparing intervention and comparison districts by chi square for contingency presentations, with r-1 x c-1 degrees of freedom where r = number of comparison groups (r = 2) and c = number of strata for each comparison (c = x), significant at: *p<0.05. **p<0.01 and ***p<0.001.

There was no difference in the distribution of women who reported giving cereals (rice, chapatti, bread bun and other cereals), irrespective of quantity and frequency, to their children from 9–18 months between the two study arms (Fig 4). The percentage of women feeding pulses and legumes was significantly higher in the intervention district at 12, 15 and 18 months, whereas feeding fruits and vegetables was significantly higher (p<0.01) in the comparison district at all ages, and ranged from 22.5% at 9 months to 67.9% at 18 months of age. The percentage of intervention district women who reported feeding fruits and vegetables ranged from 13.4% at 9 months to 55.6% at 18 months of age. Likewise, a significantly higher percentage of women in the comparison than in the intervention district reported feeding milk and milk products to their children at 12 (34.4% vs. 22.9%, p = 0.002), 15 (36.8 vs. 23.8, p = 0.001), and 18 months (44.6 vs. 35.9%, p = 0.02). Fewer than 3% of the women in either district reported feeding meat, fish or egg to their children during the first 18 months of life. And, despite the recommendation of adding oil to cooked food before serving to increase its energy density, fewer than 2% of women in either district reported adding oil to their child’s food.

### Impact on infant’s nutritional status and growth

At the first postnatal visit (<1 months), children in the intervention district were significantly shorter (49.9 ± 2.8 vs. 51.0 ± 3.1cm, p<0.007) as they were also slightly younger than the intervention district children (11.7 ± 5.7 vs. 13.6 ± 6.1 days) (Table 4). The difference diminished in the subsequent follow-up visit at 3, 6 and 9 months. At 12 month, comparison district children were marginally taller (69.5 ± 3.1 vs. 70.0 ± 3.0*, p<0.05) than their intervention district counterparts. No differences in the mean LAZ were observed for the study follow-up period, revealing no impact of the intervention on improving linear growth. The mean LAZ score at first follow-up visit also confirm that children in the intervention district, though shorter, but heavier than the children in the comparison district. Stunting prevalence did not differ between the two groups, among whom the prevalence increased dramatically between 6 and 12 months, and was highest at 15–18 months.

**Table 4:** Weight and length measurements, mean LAZ and WAZ scores and proportion of underweight and stunted infants at different ages in Uttar Pradesh, India (2004–06).

Variable description	birth to 30 days		*At 6 mo of age*		*At 12 mo of age*		*At 15 mo of age*		*At 18 mo of age*	
	(I)	(C)	(I)	(C)	(I)	(C)	(I)	(C)	(I)	(C)
Length (cm) (mean ± SD)	49.9 ± 2.8	51.0 ± 3.1**	63.8 ± 2.9	64.1 ± 3.1	69.5 ± 3.1	70.0 ± 3.0*	72.2 ± 3.2	72.6 ± 3.2	75.3 ± 3.4	75.5 ± 3.6
Weight (kg) (mean ± SD)	3.0 ± 0.6	3.1 ± 0.8	6.4 ± 0.9	6.3 ± 1.0	7.3 ± 1.1	7.3 ± 1.0	8.1 ± 1.1	8.1 ± 1.0	8.5 ± 1.1	8.6 ± 1.1
LAZ (mean ± SD)	-1.1 ± 1.1	-0.8 ± 1.2**	-1.7 ± 1.1	-1.7 ± 1.2	-2.5 ± 1.1	-2.3 ± 1.2	-2.5 ± 1.1	-2.8 ± 1.2	-2.6 ± 1.2	-2.7 ± 1.1
WAZ (mean ± SD)	-1.4 ± 1.1	-1.6 ± 1.1*	-1.8 ± 1.1	-1.9 ± 1.3	-2.3 ± 1.1	-2.5 ± 1.1	-2.0 ± 1.1	-2.2 ± 1.1	-2.0 ± 1.1	-2.1 ± 1.1
LAZ^2^ < -2.00%	18.2	16.4	38.5	37.4	69.1	59.8	67.6	75.0	69.5	72.3
WAZ^2^ < -2.00%	29.1	31.5	37.2	44.4	58.5	69.3 *	51.6	47.1	50.6	49.1

^1^ Statistical testing result comparing intervention and comparison districts by chi square for contingency presentations, with r-1 x c-1 degrees of freedom where r = number of comparison groups (r = 2) and c = number of strata for each comparison (c = x) or by t-test for comparing continuous distributions, significant at:

*p<0.05.

**p<0.01 and

***p<0.001.

^2^ Length for age z-scores and weight for age z-scores were calculated using WHO, 2006 growth standards and age in days at the time of visit

Despite being slightly younger, mean weight of intervention district children at <1 month visit was similar to the comparison district children (3.1±0.8 kg vs. 3.0±0.6 kg; p = 0.069), adjusted for age. A significantly higher mean WAZ was found in the intervention district relative to the comparison district at <1 months (-1.4 ± 0.06 vs. -1.6 ± 0.06, p<0.05) and 9 months (-2.0 ± 0.07 vs. -2.4 ± 0.09, p = 0.0026) of age. At all other ages, children from the intervention district showed a non-significant trend for higher WAZ than those from the comparison district. The only significantly lower proportion of underweight children (<-2 WAZ) in the intervention group was observed at 12 months (58.5% vs. 69.3%; p<0.05) (Table 4).

In multivariate analysis, the odds of improved breastfeeding practices were significantly increased in the intervention group, but complementary feeding practices were not. The odds of being stunted did not show any significant difference between the intervention and the comparison arms, after adjusting for baseline characteristics and neonatal visit length (Table 5). Odds of being underweight in the intervention district, though only significanlty decreased at 12 months, remained low at 6, 12 and 18 month of ages, after adjusting for baseline characteristics and neonatal visit weight.

**Table 5:** Un-adjusted and adjusted odds of reported infant feeding practices and nutritional status outcomes at different ages.

Variable	Unadjusted OR (95% CI)	Adjusted OR (95% CI)^1^
Breastfeeding practices		
Initiation of breastfeeding < 1hr	7.6 (3.9–14.9)	7.6 (3.8–15.1)
Colostrum feeding	6.2 (4.1–9.4)	7.0 (4.5–10.8)
Avoiding prelacteals	11.7 (5.6–24.4)	12.4 (5.8–26.3)
Exclusive breastfeeding at 6 mo	1.8 (1.2–2.5)	2.0 (1.4–2.9)
Complementary feeding practices		
Initiation of CF at 6 mo	1.0 (0.7–1.3)	1.0 (0.7–1.4)
At 9 mo		
Appropriate quantity	1.0 (0.7–1.5)	1.0 (0.6–1.5)
Appropriate frequency	1.1 (0.8–1.5)	1.0 (0.7–1.4)
At 12 mo		
Appropriate quantity	0.7 (0.5–1.1)	0.6 (0.4–0.98)
Appropriate frequency	1.0 (0.7–1.5)	1.0 (0.7–1.5)
At 18 mo		
Appropriate quantity	1.2 (0.8–1.8)	1.2 (0.8–1.8)
Appropriate frequency	1.4 (1.0–2.0)	1.4 (1.0–2.0)
Anthropometric measurements		
At 6 mo		
LAZ <-2 z score	1.0 (0.7–1.5)	1.1 (0.7–1.7)^2^
WAZ <-2 z score	0.7 (0.5–1.1)	0.8 (0.5–1.2)^3^
At 12 mo		
LAZ <-2 z score	1.5 (0.9–2.4)	1.7 (0.98–3.0)^2^
WAZ <-2 z score	0.6 (0.4–0.99)	0.5 (0.3–0.9)^3^
At 18 mo		
LAZ <-2 z score	0.9 (0.5–1.5)	1.2 (0.6–2.2)^2^
WAZ <-2 z score	1.1 (0.7–1.7)	1.1 (0.6–2.0)^3^

^1^ Adjusted for mother working outside, place of delivery, religion, caste and maternal age and primi birth

^2^ Adjusted for mother working outside, place of delivery, religion, caste, maternal age and height

^3^ Adjusted for mother working outside, place of delivery, religion, caste, maternal age and birth weight

A cubic spline latent growth model with knots at 6 and 18 months helped account for faster rates of growth in the first 6 months, a plateau from 6–18 months and slightly increased rates thereafter. Infants born in the intervention district increased 2.46 cm/mo in length in the first 6 months 0.84 cm/mo from 6–17 months and 0.99 cm/mo at > = 18 months, whereas the comparison district infants increased 2.36 cm/mo from 0–5 months, 0.87 cm/mo from 6–17 months and 0.89 cm/mo from > = 18 months of age. Likewise, infants in the intervention district gained 0.59 Kg/mo, 0.16Kg/mo and 0.13Kg/mo and infants in the comparison district 0.57 Kg/mo, 0.17 Kg/mo and 0.16 Kg/mo from 0–5 months, 6–17 months and > = 18 months of age respectively. Coefficients to show the differences between the intervention and comparison districts (0.13 cm/mo) suggested significant differences in length measurements at earlier ages (0–5 months). When stratified by gender, male infants in the intervention district showed a significantly higher growth rate than females in length (0.16 cm/mo) measurement. At later ages no difference by gender was observed. Furthermore, there was no difference in weight gain between the intervention and the comparison district children (Table 6).

**Table 6:** Fitted coefficients from the growth trajectory model to show the difference in the growth rate between the intervention and the comparison districts at various age interval slope estimates.

Age intervals	All		Female		Male	
	**Unadjusted** **Coef. (SE)**	**Adjusted**^1^ **Coef. (SE)**	**Unadjusted** **Coef. (SE)**	**Adjusted**^2^ **Coef. (SE)**	**Unadjusted** **Coef. (SE)**	**Adjusted**^2^ **Coef. (SE)**
**Length (cm/mo)**						
0–5 mo						
Intervention	0.12* (0.02, 0.21)	0.13** (0.03, 0.22)	0.09 (-0.03, 0.22)	0.07 (-0.05, 0.19)	0.13* (0.002, 0.25)	0.16* (0.04, 0.29)
6–17 mo						
Intervention	-0.03 (-0.08, 0.02)	-0.05 (-0.10, 0.01)	-0.01 (-0.07, 0.06)	0.001 (-0.07, 0.07)	-0.05 (-0.12, 0.02)	-0.08* (-1.15, -0.01)
≥18 mo						
Intervention	0.07 (-0.26, 0.39)	0.12 (-0.21, 0.44)	0.14 (-0.28, 0.56)	0.01 (-0.42, 0.45)	0.11 (-0.33, 0.55)	0.20 (-0.25, 0.65)
	**Unadjusted** **Coef. (SE)**	**Adjusted**^3^ **Coef. (SE)**	**Unadjusted** **Coef. (SE)**	**Adjusted**^4^ **Coef. (SE)**	**Unadjusted** **Coef. (SE)**	**Adjusted**^4^ **Coef. (SE)**
**Weight (Kg/mo)**						
0–5 mo						
Intervention	0.02 (-0.01, 0.05)	0.01 (-0.02, 0.05)	0.03 (-0.01, 0.03)	0.02 (-0.02, 0.06)	0.01 (-0.03, 0.05)	0.003 (-0.04, 0.05)
6–17 mo						
Intervention	-0.01 (-0.03, 0.01)	-0.01 (-0.03, 0.01)	-0.01 (-0.03, 0.01)	-0.01 (-0.03, 0.01)	-0.01 (-0.03, 0.02)	-0.004 (-0.03, 0.02)
≥18 mo						
Intervention	-0.03 (-0.13, 0.08)	-0.02 (-0.13, 0.08)	-0.03 (-0.17, 0.10)	-0.06 (-0.20, 0.08)	-0.001 (-0.15, 0.15)	0.02 (-0.13, 0.16)

p-value significant at:

*p<0.05,

**p<0.01,

***p<0.001.

^1^ Adjusted for gender, caste, maternal age, height, maternal education, primiparous, exclusive breastfeeding and gestational age

^2^ Adjusted for caste, maternal age, height, maternal education, primiparous, exclusive breastfeeding and gestational age

^3^ Adjusted for gender, caste, maternal age, education, primiparous birth, gestational age and reported diarrheal infection

^4^ Adjusted for caste, maternal age, education, primiparous birth, gestational age and reported diarrheal infection

## Discussion

Evaluation findings of CARE-India’s INHP II interventions in rural Uttar Pradesh demonstrate that the program was effective at improving newborn feeding practices, including increasing colostrum feeding and early initiation of breastfeeding, and reducing the provision of prelacteals. Exclusive breastfeeding at <1 months, 3 months and 6 months of age was more prevalent in the intervention than the comparison district. The intervention appeared to increase protein intake (i.e., legume consumption) among children from 9 to 18 months. However, cereal intake increased equally in both districts while intake of fruit and vegetable, milk and milk products increased more rapidly among children in the comparison than in the intervention district, for unexplainable reasons. Among children from 9 to 18 months of age, the intervention did not improve overall quantity and variety of complementary food given, and showed modest impovement in frequency only at 15 and 18 months of age.

Care India’s INHP II has been successful in improving linear growth among younger (0–5 mo) male infants. There was no significant increase in length or weight gain between the intervention and the comparison districts at later age ranges. The gender effect showed that younger male infants (0-5months) in both the intervention and the comparison districts reported better length and weight gain, however, no significant differences at the later age ranges were observed. Overall, though the intervention district children increased 0.45cm more and gained 0.06 Kg more weight per year, difference was not significant.

Though significant, but limited impact of intervention on child growth at earlier age ranges could be due to significant differences in breastfeeding initiation practices between the intervention and the comparison districts that may affect immunologic profile as there were more symptomatic infections namely, fever, diarrhea and cough/cold (data not presented). This may explain the small but significant improvement in the linear growth from 0–5 months. The consistent better growth of male infants in both the intervention and comparison districts need further explorations as we did not find any differences in infant feeding practices. There was limited evidence of a positive effect of the intervention on infant. The mean WAZ was higher at 9 months and underweight prevalence was lower among intervention children at 12 months only. The limited impact on ponderal growth only at earlier ages may not be surprising given that few, consistent differences in complementary feeding practices were observed between the two study areas. Consumption of a predominantly cereal-based diet, in small quantities, combined with a near absence of foods of animal origin in the diet, with no differences in oil added to the diet (recommended to increase the energy density) may have minimized any potential impact on child growth. Consumption of fruits and vegetable as well as milk and milk products was also higher among the comparison district children. These factors may have contributed to the overall lack of intervention impact on nutritional status in this setting. An earlier small-scale effectiveness study in a similar setting demonstrated somewhat similar results, where, despite some improvements in infant feeding practices at almost all age ranges, only a small reduction in stunting prevalence at month 18 was observed with no overall improvement in the prevalence of infant undernutrition [6].

Evaluation of INHP II faced several challenges. The phase one of the INHP was also implemented in the same district. Therefore, some residual impact of earlier program could not be ruled out. Further, since the program was already covered one district, randomization could not be done for the selection of implementation and comparison district. While the design was quasi-experimental, inability to randomize multiple program areas to either intervention or comparison status may have led to confounding that could distort findings. To this end, we attempted to achieve comparability across districts with respect to socio-demographic indicators and access to services, such as may be reflected by distance to the nearest state capital.

Analysis was based on intention to treat that does not account for heterogeneity in program implementation [19], since this analysis is the most relevant for policymakers. However, in order to understand the potential efficacy of the intervention, it will be important to look at the population that was actually exposed to the intervention and is likely to show better results. This will be reported separately and will help to understand effective components of the intervention and may help in strengthening the existing program.

The ‘CARE-enhanced’ package delivered through the system demonstrated improvement in breastfeeding practices. However, the limited and somewhat mixed effects of the intervention program on complementary feeding indicators may not be adequate to demonstrate a sustained reduction in undernutrition, especially given that increases in stunting prevalence tend to emerge during weaning. More rigorous and coordinated efforts may be required to bring about changes in complementary feeding behaviors, including repeated reinforcement for longer periods, resulting in reductions in child undernutrition scenario. Finally, given the size and complexity of the program's operations and multiplicity of outcomes across sequential age time points, it is improtant to study program exposure as it evolved over time, and components that have been successful in improving feeding practices indicators.

## Supporting information

S1 File CONSORT 2010 checklist of information to include when reporting a randomised trial. (DOC)

S2 File Cohort research protocol. (DOC)

S3 File **Complementary feeding practices at different ages in Uttar Pradesh, India (2004–06)** * Statistical test result comparing intervention and comparison districts, significant at p<0.05 **(Table A). Weight and length measurements and proportion of underweight and stunted infants at different ages in Uttar Pradesh, India (2004–06) (Table B)**.
(DOC)

## Abbreviations

ANM: Auxiliary Nurse Midwives
AWC: Anganwadi center
AWW: Anganwadi Worker
BF: Breastfeeding
CARE: Cooperative for Assistance and Relief Everywhere
CBO: Community-based Organization
CF: Complementary-feeding
Comp: Comparison
GoI: Government of India
ICDS: Integrated Child Development Services
IFA: Iron-folic acid
INHP II: Integrated Nutrition and Health Program
Int: Intervention
IYCN: Infant & Young Child Nutrition
JHBSPH: The Johns Hopkins Bloomberg School of Public Health
KGMU: King George Medical University
MoH & FW: Ministry of Health & Family Welfare
NGO: Non-government Organization
PRI: Panchayati Raj institutions
RCH: Reproductive and Child Health
WHO: World Health Organization
